# NaCl-Induced Dynamic Physiological Response and Growth Stage Sensitivity in Quinoa in Sandy Soils

**DOI:** 10.3390/plants14233639

**Published:** 2025-11-28

**Authors:** Meseret Gutema Abebe, Elizabeth Manneh, Agnes Aron Dube, Mutsa Muhambi, Mitsuru Tsubo, Kazuhiro Ujiie, Eiji Nishihara

**Affiliations:** 1 The United Graduate School of Agricultural Sciences, Tottori University, 4-101 Koyama-Minami, Tottori 680-8553, Japan; selam23.know@gmail.com (M.G.A.); elizabethmanneh19@gmail.com (E.M.); agiedube@gmail.com (A.A.D.); mutsamuhambi2022@gmail.com (M.M.); 2 Arid Land Research Center, Tottori University, 1390 Hamasaka, Tottori 680-0001, Japan; tsubo@tottori-u.ac.jp; 3 Faculty of Life and Environmental Sciences, Shimane University, 1060 Nishikawatsu-cho, Matsue 690-8504, Japan; ujiiek@life.shimane-u.ac.jp; 4 Faculty of Agriculture, Tottori University, 4-101 Koyama-Minami, Tottori 680-8553, Japan

**Keywords:** quinoa, stress, sandy soil, electrical conductivity, NaCl, leaching

## Abstract

Salinity strongly limits crop establishment in arid and semi-arid regions, yet the salinity dynamics of sandy soils, including how these dynamics relate to germination and early growth, remain poorly understood. This study integrated multi-depth, in situ bulk electrical conductivity (*ECb*) monitoring, converted to *ECe*, to quantify salinity dynamics and assess their effects on two quinoa genotypes (KD and J009) grown under freshwater (TR1), a single 200 mM NaCl pulse (TR2), or continuous 200 mM NaCl irrigation (TR3). Seed-zone salinity increased sharply following saline irrigation and declined with leaching, and actual emergence began when *ECe* dropped to approximately 8.4–11 dS m^−1^. KD showed stronger tolerance to rising salinity, maintaining ~85% emergence compared with ~55% in J009 under TR3, and exhibited 17% faster emergence and greater biomass recovery under TR2. Na^+^ accumulated mainly in leaves in KD but predominantly in stems in J009, which also experienced a 25% reduction in chlorophyll under continuous salinity, indicating greater photosynthetic inhibition. By linking dynamic EC fluctuations with genotype-specific physiological responses, this study provides a novel approach for defining salinity thresholds, identifying growth stage sensitivities, and improving salinity management in sandy, saline-prone soils.

## 1. Introduction

Water scarcity and salinity stress are major constraints to agriculture in arid and semi-arid regions, where the use of low-quality or saline water for irrigation is common [1,2]. Salt accumulation in the root zone reduces soil quality and adversely affects plant growth, highlighting the need for effective monitoring and management strategies to ensure sustainable crop production [3].

Soil salinity primarily results from the accumulation of water-soluble salts such as sodium chloride, calcium chloride, and magnesium chloride [4]. These salts dissociate into mobile ions in the soil solution and are redistributed through irrigation, leaching, and capillary rise [5]. In sandy soils with high permeability and low water retention, leaching can wash salts downward, while capillary rise can bring them back to the surface [6]. Understanding these processes is essential for mitigating salinity stress to crop production [7].

Soil electrical conductivity (EC) is a widely used indicator of salinity status because it reflects the concentration of dissolved salts in the soil solution [8]. The deployment of in situ EC sensors at multiple depths provides valuable information on salt movement within the soil profile and helps assess the effectiveness of leaching practices [8,9]. In particular, *ECb* measured by sensors such as the HydraProbe offers a practical tool for studying salinity dynamics, as it responds directly to salt redistribution in real time [10]. These continuous, multi-depth measurements make it possible to determine whether salts are displaced below the root zone during irrigation, thereby supporting data-driven irrigation scheduling and targeted salinity management strategies [11]. Moreover, previous studies have demonstrated that *ECb* thresholds can be directly applied to trigger irrigation or leaching events, effectively mitigating salinity stress and improving crop performance [12].

Quinoa (*Chenopodium quinoa* Willd.) is moderately salt-tolerant, but its sensitivity varies by growth stage, with germination being particularly vulnerable [13]. Real-time monitoring of root-zone EC allows for a precise evaluation of salinity dynamics throughout the growing season and their relationship to plant performance [9]. Salinity levels in the root zone fluctuate with irrigation frequency and water quality: infrequent irrigation increases salt concentration due to evapotranspiration, whereas frequent low-salinity irrigation can dilute and leach salts downward. Effective salinity management also requires integrating proper irrigation scheduling and leaching fractions, the portion of water draining below the root zone to remove salts [14]. Therefore, combining EC data with plant responses is essential for a comprehensive understanding of crop tolerance [15,16].

Quinoa has demonstrated the ability to grow with only limited yield loss under irrigation water salinity levels of 10–20 dS/m [17], while another study reported about 9% yield reduction at salinity levels up to 10 dS/m [18]. Interestingly, some reports have even observed improved yields within this salinity range compared to freshwater irrigation. In contrast, other findings indicate that yield begins to decline when salinity exceeds 12 dS/m [19], with a 50% reduction observed at 25 dS/m and reaching no yield at 51 dS/m [20]. These discrepancies in reported thresholds highlight the complexity of quinoa’s salinity tolerance and suggest that outcomes are strongly influenced by factors such as soil type, irrigation frequency, and salt dynamics within the root zone. Consequently, gaining a deeper understanding of salt accumulation, redistribution, and leaching under different irrigation regimes is critical to clarifying quinoa’s tolerance limits and informing management strategies for saline-prone agricultural systems.

Despite the recognized halotolerance of quinoa, the mechanisms that govern its performance under dynamic and fluctuating salinity conditions remain poorly understood—particularly in sandy soils, where rapid shifts in electrical conductivity (EC) are inherent. To address this gap, our study employs an integrated approach using multi-depth, in situ *ECb* monitoring (converted to *ECe*) to accurately track and quantify salinity dynamics within the root zone. By coupling these continuous conductivity measurements with comprehensive plant performance indicators, we aim to precisely define the dynamic salinity thresholds that limit quinoa emergence and growth and to evaluate the effectiveness of irrigation-driven leaching as a mitigation strategy. Because quinoa germination and early seedling establishment are highly sensitive to short-term changes in osmotic and ionic stress, we expect that rapid EC fluctuations will impose stronger physiological constraints than steady-state salinity of similar magnitude. We hypothesize that the transient nature of salinity in sandy soils—driven by low water-holding capacity and high leaching potential—substantially intensifies the physiological stress experienced by quinoa. Specifically, we predict that rapid EC fluctuations will exacerbate salt-induced responses, resulting in reduced seed germination, greater declines in biomass accumulation, and diminished photosynthetic capacity. Accordingly, this study seeks to clarify how dynamic salinity conditions in sandy soils influence quinoa establishment, thereby addressing a key knowledge gap in salinity–crop interactions.

## 2. Materials and Methods

### 2.1. Plant Materials, Soil Preparation, and Experimental Design

Two quinoa genotypes (KD2009_S7b and J009_S4a) were obtained from the Japan International Research Center for Agricultural Sciences (JIRCAS), Ibaraki, Japan. The greenhouse experiment was conducted at Tottori University, Tottori, Japan (35°30′ N, 134°14′ E) from early July to late October 2022.

Sandy soil was collected from the Tottori Sand Dune, thoroughly washed with water to remove soluble NaCl ions, air-dried, and sieved through a 2 mm sieve. The soil’s physical and chemical properties were analyzed prior to the experiment mentioned below: the composition was 97.2% sand, 2.8% silt, and 0% clay. The bulk density was 1.4 g cm^−3^, with a pH of 6.8 and an EC of 0.01 dS m^−1^. The total carbon (C) and total nitrogen (N) contents were 0.05 g kg^−1^ and 0.03 g kg^−1^, respectively, resulting in a C/N ratio of 1.67. Available phosphorus was 2.6 mg kg^−1^, while exchangeable potassium, calcium, and magnesium were measured at 81.4 mg kg^−1^, 63.6 mg kg^−1^, and 64.7 mg kg^−1^, respectively. The average greenhouse temperature and relative humidity were 30 ± 4 °C and 75 ± 10%, respectively. Polyvinyl chloride (PVC) pots, 25 cm in height and 15 cm in diameter, were used for the experiment. At the base of each pot, a layer of cheesecloth was placed to prevent soil loss while allowing water drainage. Each pot was filled with 6 kg of the prepared sandy soil. Pots were arranged at a heart-to-heart spacing of approximately 45 cm to minimize competition and shading among plants. The layout was randomized, and all pots received uniform light exposure. The experimental design included two quinoa (KD2009_S7b and J009_S4a), three NaCl treatments, and four replications per treatment. Ten seeds were sown per pot, with two seeds per hole. Seeds were sown at a depth of approximately 1 cm in each pot.

**Figure 1:**
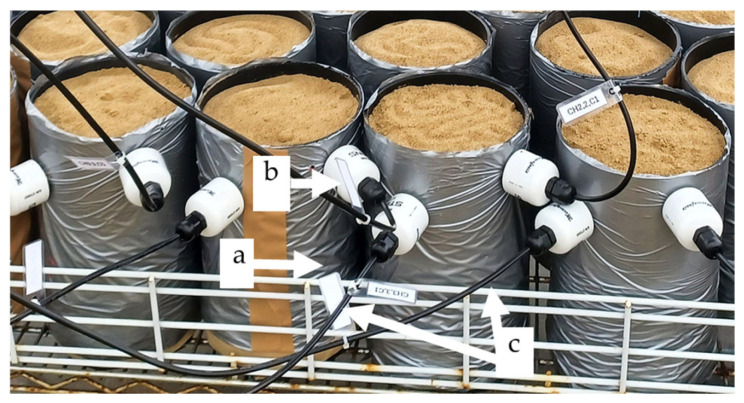
Experimental setup showing the soil-filled PVC columns used for quinoa growth under different salinity treatments. (**a**) PVC column, (**b**) HydraProbe sensor inserted at 3 cm and 6 cm depth, (**c**) sensor cable connections.

Soil moisture content and *ECb* were monitored using the Hydra Soil Moisture Probe (Stevens Water Monitoring Systems Inc., USA; hereafter referred to as “Hydra probe”) during the study. The sensors were connected to a CR1000 data logger (Campbell Scientific Ltd., Logan, UT, USA), which was programmed using PC400 software (Campbell Scientific Inc.). Irrigation was applied to maintain the soil moisture between 0.10 and 0.20 m^3^ m^−3^. For each genotype and treatment, four PVC pots were prepared (*n* = 4). One pot in each set was equipped with two Stevens HydraProbe sensors inserted horizontally at 1–3 cm and 3–6 cm depth (Figure 1); while the remaining three replicate pots were irrigated according to the real-time sensor readings from the instrumented pot. *ECb* data were normalized by correcting for temperature effects relative to 25 °C. To normalize for soil moisture and convert *ECb* to equivalent *ECe*, Equations (1) and (2) were used [21,22]. The EC of the 1:5 soil–water suspension (EC_1:5_) was measured and converted to *ECe*, which was then used to estimate the calibration constants (*a* and *b*) for converting *ECb* to *ECe*, following the method of [8].

*ECw* = *ECb*/*θ^b^* (1)

*ECe* = *a ECw* (2)

*ECw* stands for electrical conductivity of the pore water, while *ECe* represents the electrical conductivity of the soil saturated extract. The exponent *b* was determined empirically by minimizing the RMSE between measured and predicted *ECe* values. Regression of measured *ECe* against *ECw* provided the slope a (through-origin fit), which was used for subsequent ECe estimation. The calibration achieved R^2^ = 0.97 and RMSE = 0.96 dS m^−1^. The empirical exponent (*b* = 1.58) was selected. The corresponding conversion coefficient (*a* = 1.12) provided the best linear relationship between *ECw* and measured *ECe.*

### 2.2. Salt and Irrigation Treatments Applied in the Seedling Stage

The experiment included three distinct irrigation treatments applied to both quinoa genotypes (KD2009_S7b and J009_S4a). The first treatment served as the control and involved continuous irrigation with NaCl-free water throughout the experimental period (denoted as TR1). The second treatment involved a single irrigation with 200 mM NaCl solution immediately after sowing, followed by NaCl-free water for the remainder of the experiment (denoted as TR2). The third treatment involved continuous irrigation with 200 mM NaCl solution for the entire duration of the experiment (denoted as TR3).

Seedling emergence was monitored daily for 21 days. The percentage of seedling emergence and the emergence index were calculated using Equations (3) and (4), respectively, following the method of [23,24]. Fresh and dry weights of seedlings were measured after drying at 65 °C for 24 h. The mean germination time (MGT) was calculated using Equation (5) as described by [25], while the Absolute growth rate (AGR) was determined using Equation (6) according to [26]

Emergence % = *Ns*/*Stn* × 100 (3)

Emergence Index = (1/*Stn*)∑(*Et*/*Dt*) (4)

MGT = ∑ (*Et* × *Dt*)/*N* (5)

*AGR_H_* = (*Hh* − *Ht*)/*Th* − *Tt* (6)

In the equations, *Ns* represents the number of seedlings, *Stn* is the total number of seeds sown, *Et* is the number of emerged seedlings on the day *t*, *Dt* represents the number of days from the starting of the treatment, *N* is the total number of seeds germinated, *Hh* is plant height (cm) at harvest, *Ht* is plant height (cm) at transplanting, *Th* is the day of harvest, and *Tt* is the day of transplanting.

### 2.3. Salt and Irrigation Treatments Applied in the Vegetative-Inflorescence Stage

The experiment was conducted in a greenhouse at Tottori University from late August to the first week of October 2022. Quinoa seeds were initially sown in seed trays filled with sandy soil and irrigated with tap water for three weeks. The tap water used had a pH of 6.8 and an EC of 0.01 dS m^−1^. To support early seedling growth, a liquid fertilizer was applied twice per week during this period. Then, the three-week-old healthy seedlings were transplanted into individual PVC pots filled with washed sandy soil. After transplanting, no additional fertilizer was applied to prevent interference with *ECb* and moisture sensor monitoring. Each pot contained one seedling to avoid competition. Treatments began immediately after transplanting and continued for 25 days (until the onset of flowering). Soil moisture and *ECb* were continuously monitored using Hydra Soil Moisture. Irrigation was performed when the average soil moisture content dropped below 0.20 m^3^. Three distinct irrigation treatments, TR1, TR2, and TR3, were applied to both quinoa. The volume of irrigated water and the amount of Na^+^ applied in the emergence and vegetative-inflorescence stages are shown in Table 1.

#### Growth Measurements and Biomass Analysis

To assess growth performance, the length of each seedling was measured both prior to transplantation and after harvesting. Inflorescence length was also recorded in the inflorescence stage. Stem diameter was measured at the base using a digital caliper. Leaf chlorophyll content was determined in SPAD units using a SPAD-502 chlorophyll meter (Konica Minolta Sensing, Inc., Osaka, Japan). For each pot, five fully developed leaves from a single seedling were selected to obtain representative samples. After harvesting, each plant was separated into stem, leaf, inflorescence, and root components. Fresh weights were immediately recorded, followed by oven-drying at 65 °C for 24 h to determine dry biomass weights.

### 2.4. Determination of Na^+^ and K^+^ Content

Plants collected during the seedling and vegetative-inflorescence stages were separated into root, stem, leaf, and inflorescence components. Each component was oven-dried at 65 °C for 24 h and then ground into a fine powder using a mortar and pestle. The hydrochloric acid (HCl) extraction method was used for sample digestion following the procedure described by [27].

Briefly, 30 mg of the finely ground sample was placed in a test tube, and 3 mL of 1% HCl was added. The mixture was digested in a water bath at 50 °C for one hour with occasional shaking. After digestion, the solution was cooled and filtered using 60 mm filter paper (ADVANTEC). The resulting supernatant was diluted with deionized water, and the concentrations of sodium (Na^+^) and potassium (K^+^) were determined using an atomic absorption spectrophotometer (Model Z-2300, Hitachi Co., Ltd., Tokyo, Japan), and the amount is reported as mg of ions g^−1^ dry. The volume of irrigation water, total applied Na^+^, and Na^+^ levels during the time of emergence and vegetative-inflorescence stage in both quinoa KD and J009 are calculated based on Equation (7) [28,29] and presented in Table 1.

(7) $$Sodium\left(g\right)=\frac{V\times Na\times AW}{1000}$$

where *V* is the volume of irrigation water (L), and *Na* is the sodium concentration in meq/L, and *AW* is the atomic weight of sodium (23 g/mol).

### 2.5. Statistical Analysis

In this study, pots were treated as experimental units. Two fixed factors—Genotype (KD, J009) and Treatment (TR1, TR2, TR3)—were considered, including their interaction. Daily emergence counts were recorded repeatedly from each pot until emergence ceased. Data were analyzed using the general linear model (GLM) in R version 4.5.2 (R Core Team, Vienna, Austria). Model assumptions of normality and variance homogeneity were verified. Analysis of variance (ANOVA) was performed to test differences among NaCl treatments, and Tukey’s Honestly Significant Difference (HSD) test was applied at a significance level of *p* < 0.05. All other statistical analyses were conducted using IBM SPSS Statistics, version 29.0 (IBM Corp., Armonk, NY, USA).

## 3. Results and Discussion

### 3.1. The Influence of Soil EC on the Emergence of KD and J009

**Figure 2:**
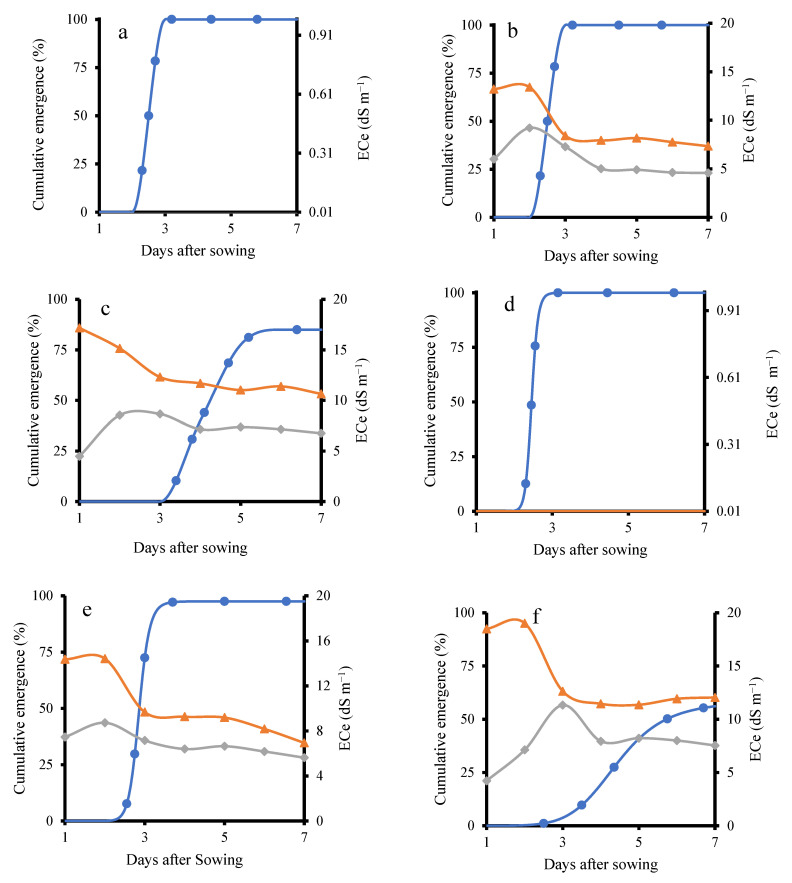
Cumulative emergence percentage and *ECe* under different treatments in KD and J009. Panels (**a**–**f**) show KD-TR1 (**a**), KD-TR2 (**b**), KD-TR3 (**c**), J009-TR1 (**d**), J009-TR2 (**e**), and J009-TR3 (**f**). Soil *ECe* at 3 cm (▲) and 6 cm (◆) depths, while emergence (%) is shown with ● symbols.

Soil bulk electrical conductivity was continuously monitored during the seedling stages using a HydraProbe sensor. For the KD cultivar, the results indicated that on the third day after sowing, the emergence percentage of the KD–TR2 treatment was 100%, matching the emergence rate of the control treatment (KD–TR1), as shown in Figure 2a,b. This occurred despite soil *ECe* levels of 8.4 dS m^−1^ at 3 cm depth and 7.2 dS m^−1^ at 6 cm depth. In contrast, the KD–TR3 treatment (Figure 2c), which received continuous irrigation with 200 mM NaCl, showed a delayed emergence. The emergence started after the third day and increased up to the sixth day to reach 85%. During this period, the *ECe* declined. The emergence started after *ECe* declined to 11 dS m^−1^. A similar finding has been reported earlier, with salinity delaying quinoa germination by up to 9 days as soil EC increased from 1 to 8 dS·m^−1^ [18].

In the KD–TR2 treatment, soil *ECe* noticeably decreased the day after switching to NaCl-free water. This decline was likely due to the downward movement of Na^+^ and Cl^−^ ions, as NaCl is highly water-soluble [30]. Successive irrigations with NaCl-free water likely flushed the dissolved salts deeper into the soil profile, thereby reducing *ECe* [31]. Similarly, in the KD–TR3 treatment, continued irrigation with saline water led to partial leaching of salts, resulting in a gradual decrease in *ECe*. In general, as shown in Figure 2, in both KD–TR2 and KD–TR3 treatments, *ECe* levels at 3 cm and 6 cm depths declined prior to seedling emergence. In both treatments, emergence was observed after the *ECe* declined to 8.4 and 11 dS m^−1^.

A trend similar to that observed in the KD cultivar was observed in J009 (Figure 2d–f), where higher salinity delayed seedling emergence. In J009–TR2, emergence reached 97% on the fourth day, coinciding with an *ECe* value of 9.6 dS m^−1^. In contrast, under the J009–TR3 condition, emergence was significantly delayed and strongly inhibited, with a maximum cumulative emergence of only 55%. These results indicate that while quinoa seeds can germinate under saline conditions, higher salinity delays or suppresses emergence, particularly in the absence of leaching [32]. Better germination is observed in KD compared with J009. This is consistent with previous studies reporting that higher NaCl concentrations reduce quinoa germination, confirming that threshold limits exist despite overall tolerance [33].

Generally, under the continuous 200 mM NaCl treatment, both quinoa cultivars showed a decline in emergence and early survival. Previous studies, however, have reported that quinoa seeds can still germinate and produce viable seedlings at 200–300 mM NaCl [34,35]. The observed complete mortality at continuous 200 mM NaCl treatment likely reflects the combined impact of salt exposure and the magnifying effect of sandy soil. The coarse texture and high drainage of the substrate would have reduced water availability at the seed–soil interface and increased osmotic/ionic stress, thereby exacerbating the inhibitory effect of salinity. This is consistent with previous findings in quinoa, where coarse-textured soils magnified salinity stress during germination and early growth [36].

### 3.2. Salinity Effects on Quinoa Germination and Early Growth

**Figure 3:**
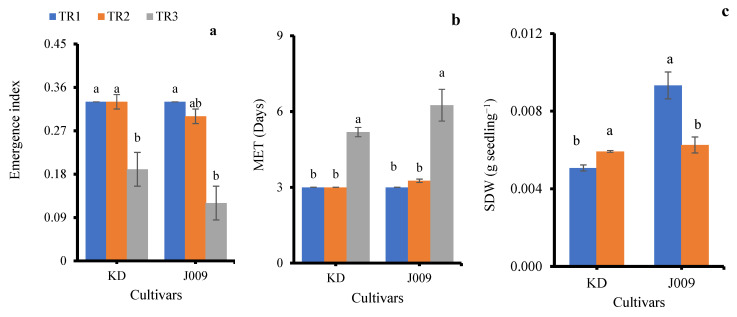
Emergence index (**a**), mean emergence time (**b**), and seedling dry weight (**c**) of KD and J009 under treatments of TR1, TR2, and TR3. Bars = mean ± SE (*n* = 4). Different letters indicate significant differences within genotype at *p* < 0.05.

The emergence index (EI) was significantly affected by salinity treatments in both cultivars (Figure 3a). Under freshwater irrigation (TR1), KD and J009 showed the highest EI (0.33), indicating rapid and uniform seedling emergence. When fresh water was replaced with continuous saline irrigation (TR3), EI markedly decreased in both cultivars (0.19 in KD and 0.12 in J009; *p* < 0.05), demonstrating the inhibitory effect of persistent NaCl stress [30]. The higher EI under TR2 suggests that post-sowing freshwater flushing alleviates early salt stress and promotes emergence.

Mean emergence time (MET) was strongly affected by irrigation regimes (Figure 3b). Under freshwater (TR1) and TR2 (initial saline then freshwater), MET remained around 3 days in both cultivars, showing that leaching effectively alleviated salinity stress. In contrast, continuous saline irrigation (TR3) significantly delayed emergence, increasing MET to ~5 days in KD and ~6 days in J009. Overall, continuous salinity slowed seedling establishment, whereas leaching restored emergence rates to near control levels.

Seedlings from both KD–TR3 and J009–TR3 treatments did not survive beyond 15 days; therefore, shoot dry weight (SDW) data were not available for these treatments. Among the remaining treatments, SDW was significantly influenced by the irrigation regime (Figure 3c). In KD, SDW increased under the TR2 treatment by ~15% compared with the control, suggesting a positive recovery response when saline irrigation was replaced with freshwater. In contrast, J009 exhibited a 33% reduction in SDW under the TR2 treatment. Previous studies have shown that biomass declines with increasing soil EC [37]. This reduction may result from the higher energy demand for osmotic regulation and osmolyte synthesis during growth [38], as well as Na^+^ toxicity in leaves and stems that disrupts enzyme activity, damages cell membranes, and interferes with nutrient uptake [33,39]. Both processes likely contributed to the growth reduction observed under salinity stress.

### 3.3. Na^+^ and K^+^ Accumulation and Ion Balance in Seedlings

**Figure 4:**
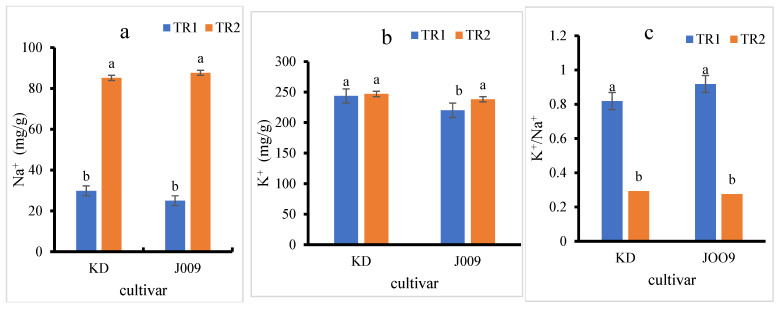
Sodium (Na^+^) (**a**), potassium (K^+^) (**b**), and K^+^/Na^+^ ratio (**c**) in KD and J009 quinoa seedlings under different treatments. Different lowercase letters indicate significant differences between treatments within each genotype at *p* < 0.05. Values are mean ± SE (*n* = 4).

As shown in Figure 4a, the Na^+^ content in the seedlings of both quinoas increased approximately threefold under the TR2 treatment compared to the control. In contrast, the K^+^ content remained relatively constant in the KD cultivar, while a slight increase was observed in J009 (Figure 4b). However, the K^+^/Na^+^ ratio decreased sharply in the TR2 treatment for both quinoa, indicating a disruption in ion homeostasis under saline conditions, as shown in Figure 4c [40]. The sharp decline in the K^+^/Na^+^ ratio under the TR2 treatment indicates that both cultivars had a reduced capacity to maintain ionic balance when exposed to NaCl. This imbalance reflects excessive Na^+^ accumulation in tissues with only limited adjustment in K^+^ levels, leading to ionic toxicity. Such disruption can impair enzyme activity, interfere with protein synthesis, reduce photosynthetic efficiency, and hinder osmotic regulation.

### 3.4. Effect of NaCl Treatment on the Vegetative-Inflorescence Stage Characteristics

**Figure 5:**
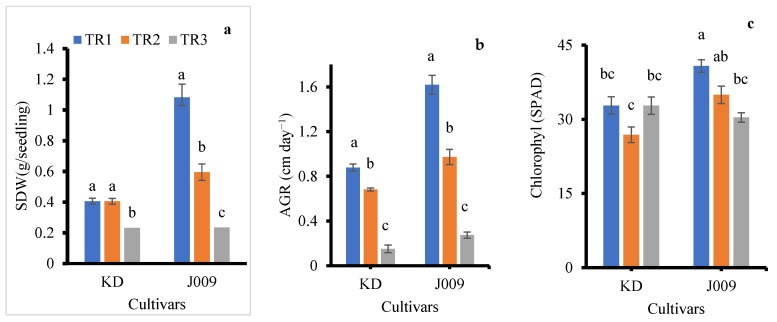
Effect of NaCl treatments on shoot dry weight (**a**), growth rate (**b**), and chlorophyll content (**c**) during the vegetative-inflorescence stage. Bars represent mean ± SE (*n* = 4), and different letters indicate statistically significant differences at *p* < 0.05.

The measurements revealed that irrigation treatments had a clear impact on vegetative-inflorescence growth characteristics, as summarized in Table 2 and Table S1. Overall, the growth rate was significantly influenced by salinity levels. Seedlings irrigated continuously with NaCl-free water exhibited the most robust growth. In contrast, as shown in Figure 5a, shoot dry weight declined with increasing NaCl concentration, consistent with previous reports that salinity stress significantly reduced plant biomass [41]. Notably, plants that received a single irrigation with 200 mM NaCl followed by NaCl-free water showed better growth performance than those subjected to continuous irrigation with 200 mM NaCl. This might be because of the partial salt leaching from the root zone, thereby mitigating the adverse effects of salinity on growth.

**Figure 6:**
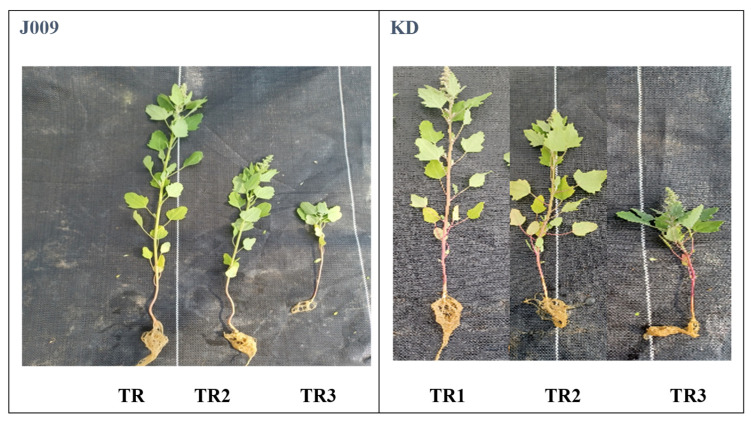
Representative KD and J009 quinoa plants initially germinated under non-saline conditions, transplanted after three weeks, and then exposed to different salinity treatments in the vegetative-inflorescence stage.

The control treatment produced the longest inflorescence in both accessions and cultivars (Table S1). An increase in salinity has been reported to reduce key yield-related traits, such as the number of leaves and the length of each panicle [42]. A significant reduction in absolute growth rate was observed in both accessions and cultivars as NaCl levels increased (Figure 5b). Plant height, stem diameter, and leaf number were all negatively affected by salinity in both treatments for both cultivars, as shown in Table 2 and Table S1 [19,43]. Thus, salinity inhibited both leaf initiation and expansion, resulting in smaller canopies (Figure 6). The dry matter percentage (DM%) increased with increasing salinity from TR2 to TR3, and the increase was more pronounced in cultivar J009. This trend indicates a reduction in tissue water content under salt stress. Among the plant organs, stems generally exhibited higher DM% than leaves and inflorescence (Table 2).

Leaf chlorophyll content was affected by NaCl treatment in both quinoa accessions and cultivars, as shown in Figure 5c. In the J009 cultivar, chlorophyll content decreased under both salinity treatments (TR2 and TR3) compared to the control [44]. The highest chlorophyll level was recorded in the control group, while the lowest was observed in the TR3 treatment. In contrast, for the KD cultivar, the lowest chlorophyll content was found in the TR2 treatment, indicating a different physiological response to the timing and duration of salt stress. The better chlorophyll retention in KD reflects a tolerance strategy and likely resulted from stronger vacuolar compartmentalization. Efficient Na^+^ sequestration within leaf vacuoles helped maintain osmotic balance and prevent ion toxicity in chloroplasts, thereby supporting sustained photosynthetic activity under salinity [43]. Growth was significantly inhibited by the presence of Na^+^ in the soil. Figure 6 further supports this observation, showing that the growth of quinoa accessions and cultivars during the vegetative-inflorescence stage was adversely affected by both salinity treatments compared to the control. Even a single application of 200 mM NaCl led to reduced growth parameters.

Representative plants of J009 and KD under different salinity regimes are shown in Figure 6. Continuous exposure to 200 mM NaCl (TR3) markedly reduced growth, whereas plants in the TR2 treatment showed partial recovery compared with the control. These visual differences are consistent with the biomass and *ECb*/*ECe* data, confirming that leaching alleviates salt accumulation in the seed zone and improves plant performance. The growth reduction observed under continuous salinity (TR3) is likely linked to impaired energy metabolism, as prolonged salt stress has been shown to downregulate photosynthesis-related genes and disrupt starch and sucrose metabolism, thereby limiting the energy supply required for growth [33].

In general, KD exhibited greater stability under salinity treatment compared to J009. During the seedling stage, KD showed higher salt tolerance, with 17% faster emergence, a 14% increase in seedling biomass under the TR2 treatment, and higher chlorophyll retention under continuous salinity (Figure 2, Figure 3c and Figure 5c). In contrast, J009 showed delayed emergence and a 33% reduction in seedling biomass. During the vegetative-inflorescence stage, J009 showed higher absolute growth rate and shoot dry weight (Figure 5a,b); however, both traits declined more sharply relative to the control, indicating lower stability under salinity (Figure 5). Conversely, KD maintained stable chlorophyll content and experienced smaller reductions in dry weight and growth rate, suggesting stronger physiological tolerance despite its lower absolute biomass. Overall, these results indicate that KD maintains greater functional stability under saline conditions, while J009 is more sensitive to salinity stress.

Comparing the effects of salinity imposed during the seedling phase and the vegetative-inflorescence phase revealed that the seedling phase was considerably more sensitive, resulting in significant reductions in emergence rate, early growth, and plant establishment (Figure 2 and Figure 3). This confirms that quinoa plants experience the greatest stress during the initial establishment phase, while tolerance increases at later developmental stages.

### 3.5. The Distribution of Ions in Plant Organs Under NaCl Stress

**Figure 7:**
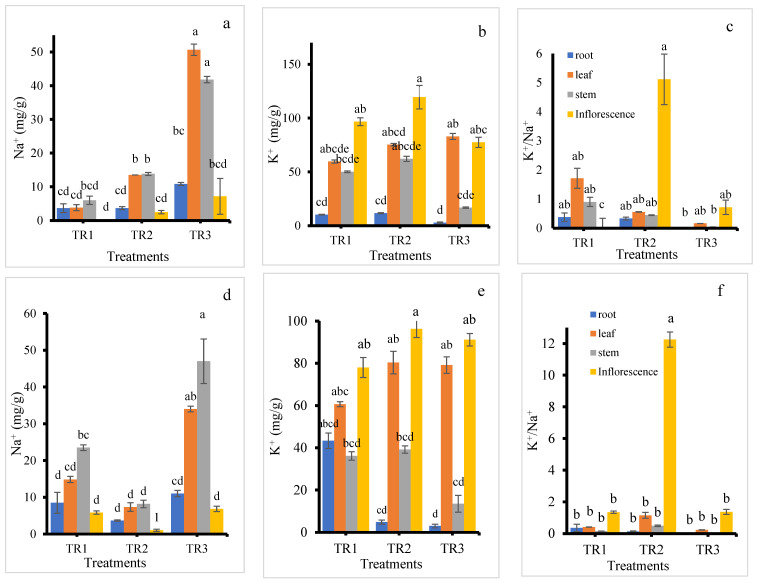
Na^+^ content (**a**,**d**), K^+^ content (**b**,**e**), and K^+^/Na^+^ ratio (**c**,**f**) in the roots, stems, leaves, and inflorescence of KD (**a**–**c**) and J009 (**d**–**f**) quinoa under different NaCl treatments. Different letters indicate significant differences between treatments within the same plant part at *p* < 0.05, mean ± SE (*n* = 4).

In the KD cultivar, Na^+^ content increased in all plant parts with increasing NaCl treatment levels compared to the control (Figure 7a). The highest accumulation of Na^+^ was observed under the TR3 treatment across all plant parts. Among the organs, the inflorescence consistently exhibited the lowest Na^+^ content. In the TR2 treatment, Na^+^ levels were similar in both the leaf and stem. Under the TR3 treatment, leaves showed the highest Na^+^ accumulation, followed by stems. Leaching (TR2) reduced this effect. It is reported that salinity promotes sodium buildup in quinoa leaves, indicating that foliage acts as a major Na^+^ sink [39,45].

K^+^ content in the roots was consistently low across all treatments, as shown in Figure 7b. The inflorescence had the highest K^+^ accumulation, with the maximum observed in the TR2 treatment. Interestingly, under the TR3 treatment, K^+^ content in the leaf was also relatively high. K^+^ accumulation in the leaf increased with rising NaCl levels. The inflorescence in the TR2 treatment exhibited the highest K^+^/Na^+^ ratio, while the control inflorescence showed the lowest ratio. These results suggest that K^+^ allocation in quinoa is organ-specific. The consistently low K^+^ in roots may indicate limited storage belowground, with preferential translocation to metabolically active organs such as inflorescence and leaves. The high K^+^ content and elevated K^+^/Na^+^ ratio in inflorescence under the TR2 treatment imply that leaching with NaCl-free water after initial salt exposure helped maintain favorable ionic balance during reproduction, which is critical for pollen viability and seed set.

In the J009 cultivar, inflorescence also maintained lower Na^+^ levels across all treatments compared to other plant parts, whereas the stem recorded the highest Na^+^ content (Figure 7d). The TR3 treatment resulted in the highest Na^+^ accumulation in all plant parts, while the TR2 treatment showed the lowest. K^+^ content was consistently higher in the inflorescence under all treatments. The roots in both TR2 and TR3 treatments had the lowest K^+^ levels, as shown in Figure 7e. In contrast, leaf, stem, and inflorescence tissues in the TR2 treatment recorded the highest K^+^ concentrations. Interestingly, the control root showed the highest K^+^ content among root samples across treatments. The inflorescence displayed a higher K^+^/Na^+^ ratio than other plant parts, with the highest value recorded in the TR2 treatment. Under the TR3 treatment, KD showed greater Na^+^ accumulation in the leaf, whereas J009 accumulated more Na^+^ in the stem. However, a similar trend was observed in the K^+^/Na^+^ ratio across both cultivars—inflorescence from the TR2 treatment consistently exhibited the highest K^+^/Na^+^ ratios, as shown in Figure 7c,f, indicating better ion balance in reproductive tissues under moderate salinity stress.

In both quinoa cultivars, roots maintained low Na^+^ levels but transported most of it to the shoots, leading to high Na^+^ accumulation in leaves and stems. These results suggest that ion toxicity, resulting from excessive Na^+^ accumulation in aerial tissues, is a more significant contributor to salt stress in these quinoa cultivars than osmotic stress. This finding was consistent with the result of [46], who reported that quinoa exhibits a Na^+^ includer strategy, with sodium predominantly accumulated in the shoots. Additionally, inflorescence in both cultivars showed much higher K^+^ concentrations than Na^+^, which may play a crucial role in supporting seed development under saline conditions. The elevated K^+^ content observed under the TR2 treatment suggests that K^+^ uptake was enhanced by moderate Na^+^ supply [47]. However, under continuous salinity (TR3), K^+^ uptake declined in most tissues, except for the leaves. This pattern supports previous findings that K^+^ accumulation in leaf tissues contributes significantly to osmotic adjustment under high salinity conditions. Between the two treatments, TR2 had a less detrimental effect on plant growth compared to TR3, reinforcing the importance of leaching or dilution of salts after initial exposure.

At the whole-plant level, quinoa appears to protect younger leaves and upper plant parts from excessive Na^+^ by compartmentalizing the ion [34,48]. However, despite protective mechanisms, significant Na^+^ accumulation occurred in leaves and stems rather than being retained in the roots, likely due to insufficient Na^+^ exclusion at the root level [49]. The Na^+^ concentration was determined in the stem of KD under treatment TR3, and the measured value (73.1 mM) was considerably lower than the external treatment concentration (200 mM) [28]. In quinoa, limited sequestration capacity in root vacuoles and strong xylem loading can cause most absorbed Na^+^ to move upward, leading to higher accumulation in photosynthetic tissues [44]. As an essential cation for plant growth, K^+^ plays a vital role not only as an enzyme cofactor but also in maintaining vacuolar osmotic potential. It contributes to the maintenance of a high cytosolic K^+^/Na^+^ ratio, which is a key determinant of salt tolerance [50]. The observed patterns in ion distribution underscore quinoa’s complex salt stress response, combining ion exclusion, compartmentalization, and selective ion uptake to support growth and reproduction under saline conditions.

### 3.6. Vertical NaCl Distribution and Soil ECe Dynamics

**Figure 8:**
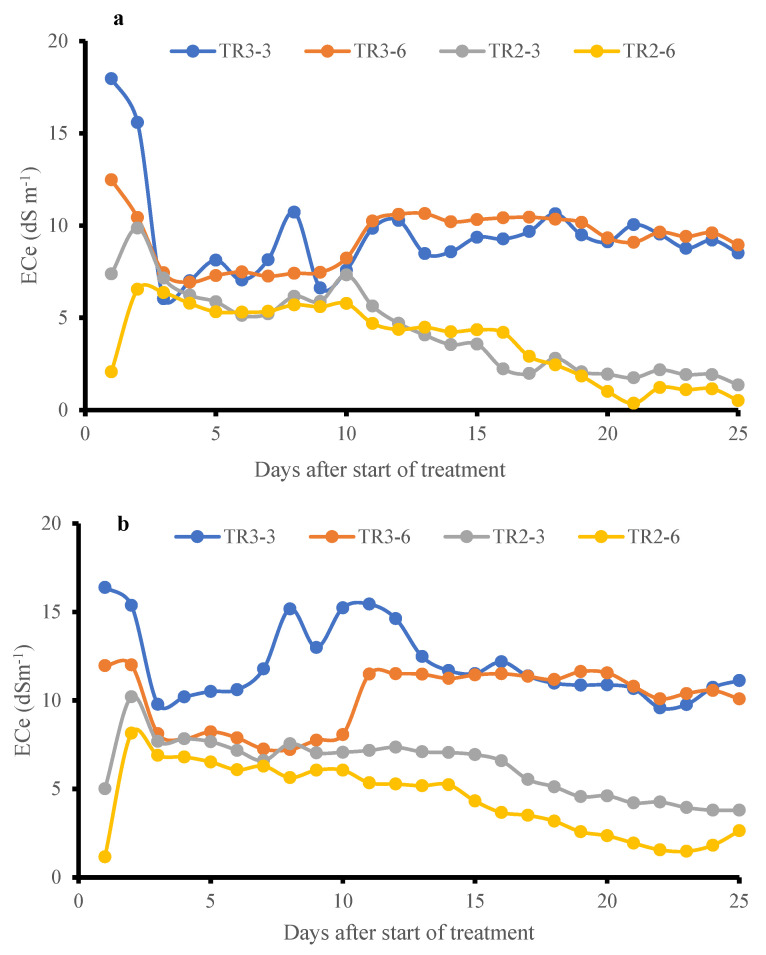
Temporal variation in soil ECe at 3 cm and 6 cm depths under TR2 and TR3 treatment for J009 (**a**) and KD (**b**) quinoa during the vegetative-inflorescence stage. Data represents changes from day 21 to 46 during the vegetative-inflorescence stage.

The effect of salt distribution was examined by monitoring soil ECe at different depths within the pots [9]. Since NaCl dissociates into Na^+^ and Cl^−^ ions in water-saturated soil, the presence of these ions proportionally increases soil conductivity. As shown in Figure 8a,b, soil ECe in both KD and J009 cultivars was high during the initial saline irrigation of TR3, then declined as dilution and leaching occurred, followed by a slight rise before stabilizing. The upper soil layer (3 cm) initially showed higher ECe than the lower layer (6 cm). For KD during TR3, ECe at 6 and 3 cm became equivalent starting from the 14th day, indicating downward salt movement; similarly, in the case of J009, it started on the 17th day. These results suggest active leaching even under continuous saline irrigation (TR3) and highlight the dynamic redistribution of salts in the sandy soil profile [51]. The stabilization of soil EC under continuous saline irrigation may be attributed to a dynamic equilibrium between the NaCl in the soil and the downward-moving irrigation water, which gradually reached a balance in salt concentration, preventing further ECe increase [51]. These observations highlight the importance of leaching dynamics in managing soil salinity and suggest that even under high salinity input, salt concentration in sandy soils can stabilize due to natural drainage and capillary action [5]. Despite uniform saline irrigation being adopted across the cultivars, slight variations in ECe were observed. These differences likely reflect minor fluctuations in micro-scale heterogeneity within the sandy substrate. Such variability is inherent to sandy soils owing to their high hydraulic variability and spatial heterogeneity.

## 4. Conclusions

This study confirmed that the transient salinity dynamics of sandy soils strongly influence quinoa emergence and early growth. Emergence occurred only when seed-zone ECe decreased to approximately 8.4–11 dS m^−1^, indicating a critical salinity range for germination under the present conditions. Continuous saline irrigation maintained elevated ECe levels, delaying or suppressing emergence, whereas leaching with NaCl-free water effectively displaced salts below the root zone and improved growth performance. Although both emergence and vegetative-inflorescence stages were affected by salinity, the seedling phase was substantially more sensitive, with early exposure causing pronounced reductions in emergence and early growth. The shoot growth and biomass accumulation were also negatively affected by elevated salinity. Genotype responses differed markedly: KD tolerated rising salinity more effectively, maintaining higher emergence and showing better recovery, whereas J009 exhibited greater chlorophyll loss and more adverse physiological responses under continuous salinity. These results highlight the value of selecting more salinity-tolerant genotypes such as KD, applying timely freshwater leaching, and using EC-based monitoring to optimize irrigation in sandy, saline-prone soils. Understanding these short-term salinity dynamics is therefore critical for improving crop establishment in arid and semi-arid regions dominated by sandy soils.

## Figures and Tables

**Table 1:** Irrigation volume and Na^+^ content in KD and J009 quinoa under different treatments and growth stages.

Growth Stage	Cultivar	Pot-Treatment Level	Volume of Irrigated Water (L) per Pot During the Treatment	Amount of Na^+^ (g) in Irrigated Water per Pot During the Treatment	Amount of Na^+^ (g) Applied per Pot During Emergence
Seedling	KD	TR1	1.17	–	–
		TR2	0.85	0.64	0.64
		TR3	0.69	3.2	1.84
	J009	TR1	1.285	–	–
		TR2	0.74	0.64	0.64
		TR3	0.7	3.2	1.88
Vegetative-inflorescence stage	KD	TR1	2.38	–	–
		TR2	2.12	0.64	–
		TR3	1.07	4.922	–
	J009	TR1	2.23	–	–
		TR2	1.75	0.64	–
		TR3	0.97	4.462	–

**Table 2:** Dry weight and dry matter percentage of leaf, inflorescence, and stem of two quinoa cultivars under different treatments. Different letters indicate significant differences at *p* ≤ 0.05, mean ± SE (*n* = 4).

Genotype	Treatments	Leaf Dry Weight (g.seedling^−1^)	Leaf Dry Matter %	Inflorescence Dry Weight (g.seedling^−1^)	Inflorescence Dry Matter %	Stem Dry Weight (g.seedling^−1^)	Stem Dry Matter %
KD	TR1	0.23 ± 0.02 ^a^	15.75	0.05 ± 0.003 ^a^	13.51	0.13 ± 0.01 ^a^	17.80
	TR2	0.26 ± 0.02 ^a^	10.56	0.04 ± 0.003 ^b^	12.90	0.11 ± 0.01 ^a^	14.28
	TR3	0.16 ± 0.01 ^b^	11.85	0.04 ± 0.002 ^b^	17.39	0.04 ± 0.003 ^b^	19.04
J009	TR1	0.61 ± 0.08 ^a^	13.99	0.13 ± 0.02 ^a^	15.47	0.34 ± 0.02 ^a^	14.53
	TR2	0.34 ± 0.05 ^b^	11.56	0.07 ± 0.006 ^b^	14.0	0.18 ± 0.02 ^b^	14.63
	TR3	0.14 ± 0.01 ^b^	12.38	0.044 ± 0.0042 ^b^	15.71	0.053 ± 0.004 ^b^	20.38

## Data Availability

All data supporting the findings of this study are included within the manuscript. Additional datasets or analysis files are available from the corresponding author upon reasonable request.

## Supplementary Materials

The following supporting information can be downloaded at https://www.mdpi.com/article/10.3390/plants14233639/s1.
