# Supplementary material for: NaCl-Induced Dynamic Physiological Response and Growth Stage Sensitivity in Quinoa in Sandy Soils
Source: Plants (Basel). 2025 Nov 28;14(23):3639. doi: 10.3390/plants14233639 (PMC12694062; doi:10.3390/plants14233639)
Supplement: Supplementary file 1 [file plants-14-03639-s001.zip › plants-3939273-supplementary.pdf]

Supplementary materials

Table S1. Growth characteristics and organ fresh weights of two quinoa cultivars under different salt treatments in the vegetative-inflorescence stage. Different letters indicate significant differences at $p \leq 0.05$ (one-way ANOVA).

Genotype	Treatments	Growth parameter						
		Leaf number	Stem diameter (⌀)	Shoot length (cm)	Inflorescence length (cm)	Fresh weight		
						Leaf ($g.seedling^{-1}$)	Inflorescence ($g.seedling^{-1}$)	Stem ($g.seedling^{-1}$)
KD	TR1	$20.0±0.71^{a}$	$1.33±0.11^{a}$	$24.9±0.83^{a}$	$3.13±0.13^{a}$	$1.46±0.09^{b}$	$0.37±0.02^{a}$	$0.73±0.05^{a}$
	TR2	$23.0±1.47^{a}$	$1.40±0.07^{a}$	$21.1±0.38^{b}$	$2.88±0.125^{a}$	$2.46±0.14^{a}$	$0.31±0.02^{ab}$	$0.77±0.04^{a}$
	TR3	$15.5±0.5^{b}$	$0.86±0.082^{b}$	$12.3±0.32^{c}$	$2.75±0.14^{a}$	$1.35±0.09^{b}$	$0.23±0.02^{b}$	$0.21±0.01^{b}$
J009	TR1	$43.5±2.33^{a}$	$2.19±0.11^{a}$	$39.6±1.42^{a}$	$5.37±0.52^{a}$	$4.36±0.65^{a}$	$0.84±0.12^{a}$	$2.34±0.33^{a}$
	TR2	$27.0±3.39^{b}$	$1.64±0.09^{b}$	$27.4±0.85^{b}$	$3.62±0.24^{b}$	$2.94±0.43^{a}$	$0.5±0.05^{b}$	$1.23±0.23^{b}$
	TR3	$15.3±0.85^{c}$	$0.99±0.12^{c}$	$15.8±0.43^{c}$	$2.75±0.14^{b}$	$1.13±0.07^{b}$	$0.28±0.02^{b}$	$0.26±0.03^{c}$
